# Biallelic *MYH3* Variants Cause Distal Arthrogryposis in Compound Heterozygosity and a Subclinical Phenotype in Simple Heterozygosity. Codominance or Recessive Inheritance?

**DOI:** 10.1111/cge.70142

**Published:** 2026-01-21

**Authors:** Omar Zgheib, Thomas Rio‐Frio, Michel Guipponi, Thierry Nouspikel, Siv Fokstuen, Marc Abramowicz, Philippe Khau Van Kien

**Affiliations:** ^1^ Service de Médecine Génétique Hôpitaux Universitaire Genève Genève Switzerland; ^2^ Département de Médecine Génétique et Développement, Faculté de Médecine Université de Genève Genève Switzerland; ^3^ Universitäres Herzzentrum Zürich, Klinik für Kardiologie Universitätsspital Zürich Zürich Switzerland

**Keywords:** distal arthrogryposis, mode of inheritance, *MYH3* gene

## Abstract

Distal arthrogryposis constitutes a highly heterogeneous group of disorders with a critical need for clear classification. Phenotypes have traditionally been characterized using the classification system proposed by Bamshad or Hall for distal arthrogryposis. Recessive *MYH3* inheritance has been described in contractures, pterygia and spondylocarpotarsal fusion syndrome, and, more recently, in distal arthrogryposis without skeletal fusion. We hereby report a nuclear family affected by distal arthrogryposis with biallelic *MYH3*‐related disorder, identifying two novel variants, which in the heterozygous state yield a subclinical phenotype. Beyond refining the molecular diagnosis and guiding genetic counseling, our study emphasizes that the classification of *MYH3*‐related disorders and their inheritance modes is still evolving, underscoring the need to integrate knowledge gained from careful analyses.

## Graphical abstract

A nuclear family affected by distal arthrogryposis with novel biallelic *MYH3* variants, which at the heterozygous state yield a subclinical phenotype, highlighting the complexity of *MYH3*‐related disorders and their inheritance modes.

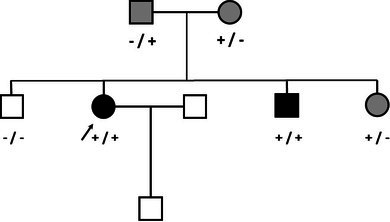

## 1 Introduction

Distal arthrogryposis multiplex congenita (AMC), also known as multiple congenital contractures (MCC), constitutes a highly heterogeneous group of disorders, highlighting the critical need for clear classification [1]. Among AMC, the spectrum of phenotypic abnormalities associated with molecular defects in the myosin heavy chain 3 gene (*MYH3*) is quite complex. These phenotypes have traditionally been described using the classification system initially proposed by Bamshad for distal arthrogryposis (DA) [2]. Types 2A and 2B3 (Freeman–Sheldon syndrome, MIM#193700 and Sheldon–Hall syndrome, MIM#618436, respectively) are caused by autosomal dominant *MYH3* mutations. Type 8 (formerly Multiple pterygium syndrome) is now designated as Contractures, pterygia and spondylocarpotarsal fusion syndrome type 1A (CPSFSIA, MIM#178110) or type 1B (CPSFSIB, MIM#618469). These are characterized by dominant and recessive *MYH3* inheritance, respectively, the latter identified in 2018 and not included in the initial Bamshad classification [3, 4, 5].

The widespread use of genomic techniques to explore arthrogryposis, such as whole‐exome and whole‐genome sequencing, has occasionally uncovered rare clinical or familial presentations that do not fit the Bamshad or Hall classifications [5].

We hereby report a nuclear family affected by AMC with biallelic *MYH3*‐related disorder, identifying two novel variants, which at the heterozygous state yield a subclinical phenotype.

## 2 Case Report

**FIGURE 1:**
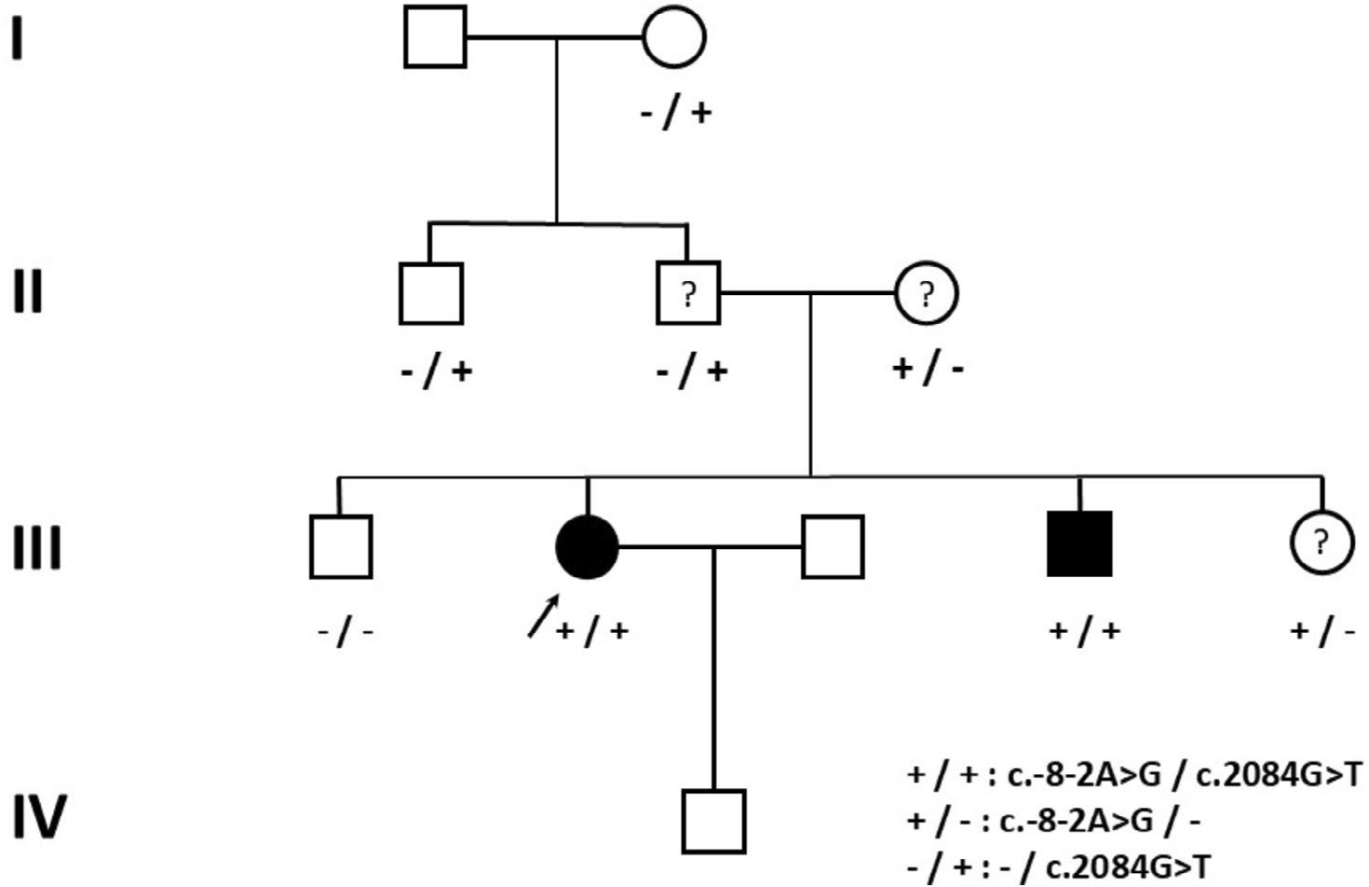
Pedigree with indication of phenotype (individuals with full distal arthrogryposis are indicated by filled symbols; those with suggestive signs, by a question mark) and genotype (indicated as +/− for the c.‐8‐2A>G and −/+ for the c.2084G>T variant) where known.

**FIGURE 2:**
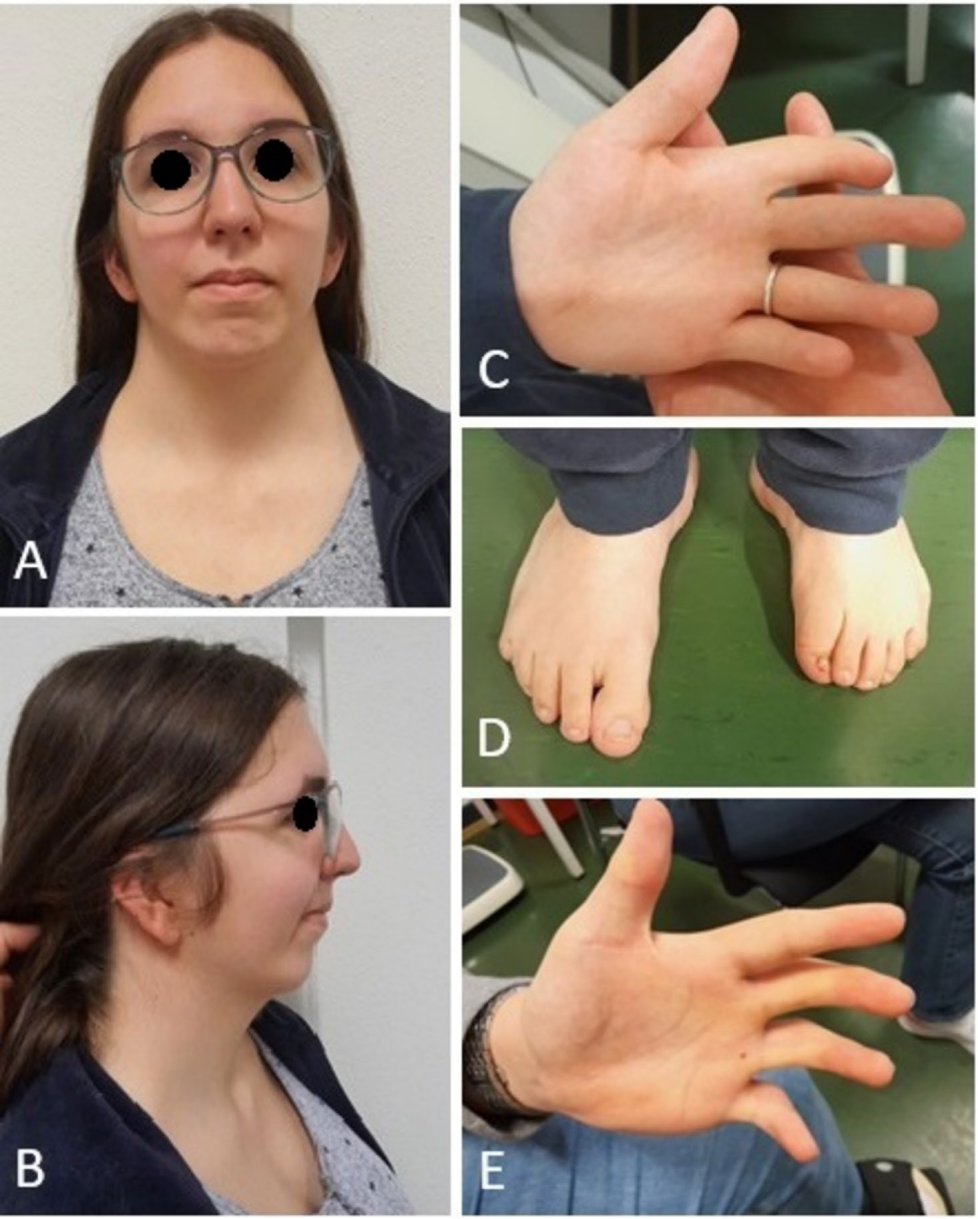
Patient photographs. (A) Portrait of proband showing webbed neck, low‐set ears, and downward‐slanting palpebral fissures. (B) Profile of proband showing retrognathia and low‐set ears with folded, hypoplastic helices and voluminous lobes. (C) Proband hand with (bilateral) camptodactyly sparing the thumbs, webbed fingers (proximal syndactyly), and absence of palmar creases and dermatoglyphs. (D) Proband feet showing sequelae of surgical interventions for bilateral clubfeet. (E) Hand of the proband's affected brother showing camptodactyly and absence of palmar creases and dermatoglyphs. [Colour figure can be viewed at wileyonlinelibrary.com]

The proband (Individual III.2) was a 20‐year‐old woman with normal intellectual abilities known for AMC with multiple operations for bilateral clubfeet, camptodactyly, and spina bifida (Figures 1 and 2A–D). She presented with a webbed neck (pterygium colli), a very low posterior hairline, low‐set ears with folded, hypoplastic helices, and voluminous lobes, downward‐slanting palpebral fissures, retrognathia, moderate bilateral camptodactyly sparing the thumbs, webbed fingers (proximal syndactyly), and absence of palmar creases and dermatoglyphs (Figure 2A–C).

She had a similarly affected brother (III.3), who presented with DA with operated bilateral clubfeet, severe camptodactyly, webbed fingers (proximal syndactyly), contractures in the elbows, wrists, and shoulders, and rotational limitation of the cervical vertebrae. He also showed absence of palmar creases and dermatoglyphs (Figure 2E), low posterior hairline, and low‐set ears.

**FIGURE 3:**
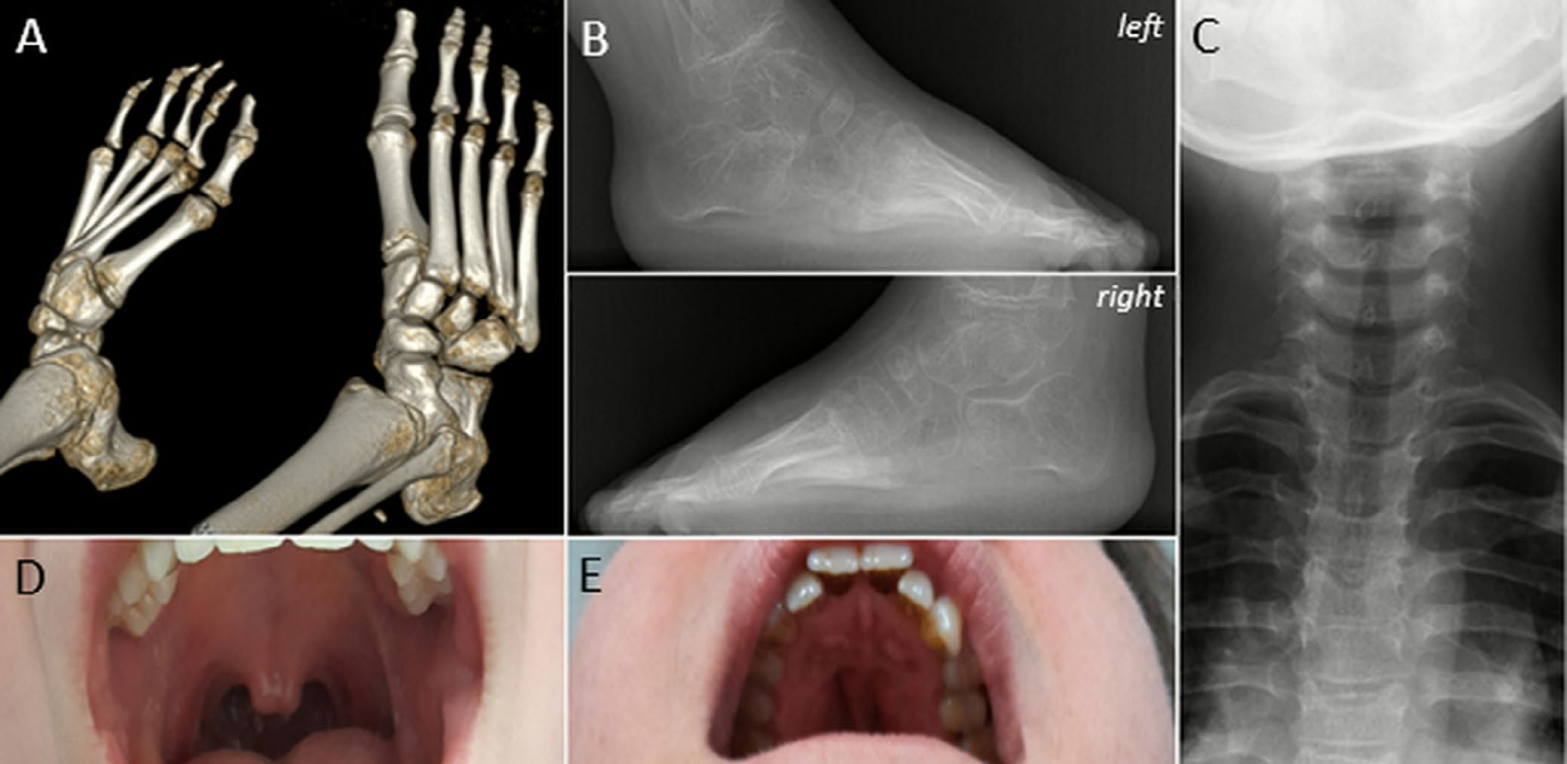
(A) Foot CT scan reconstruction (proband at age 11); (B, C) Foot and cervicothoracic spine radiographs (brother at age 6) showing absence of skeletal fusion. (D) Bifid uvula and a discrete posterior submucosal cleft palate in the sister. (E) Sequelae of cleft palate surgery in the mother. [Colour figure can be viewed at wileyonlinelibrary.com]

Neither the proband nor her brother had scoliosis; radiographs and CT scans did not identify spondylocarpotarsal or other skeletal fusions (Figure 3A–C).

There were two other siblings: an unaffected brother (III.1) and a sister (III.4) who presented with only a bifid uvula and a discrete posterior submucosal cleft palate that could belong to the AMC spectrum of manifestations (Figure 3D).

The mother (II.3) was known for bilateral hip dysplasia and cleft palate repair surgery at 18 months of age without any other clinical signs of AMC (Figure 3E). The father (II.2) was in apparent good health but reported retraction of the toes and a feeling of stiffness in the upper and lower limb muscles, “loosening up” with physical activity. There were no other clinical signs or symptoms related to AMC. We further obtained samples for the paternal uncle (II.1) and grandmother (I.2), both presumably asymptomatic but whom we were unable to examine.

The proband had been clinically diagnosed several years ago with type 2B arthrogryposis (Sheldon‐Hall syndrome) and sought genetic counseling in the context of an unexpected pregnancy, given that her condition had been assumed to follow autosomal dominant inheritance, with variable expressivity (supposing maternal inheritance). Indeed, hip dysplasia and cleft palate had been known to be associated with certain forms of AMC and considered as possible mild or subclinical manifestations. At that time, no genetic tests for molecular diagnosis had been proposed.

We thus performed whole exome sequencing in our proband targeting a DA panel of 186 genes. Genomic DNA was extracted from whole venous blood. Exome capture and sequencing were performed using the Twist Human Comprehensive Exome kit (Twist Bioscience) and a NextSeq500 sequencer (Illumina), respectively, followed by targeted panel and mendeliome analysis. Whole exome coverage was 99.2% at 20× depth. Variants were searched for in various databases including Genome Aggregation Database, ClinVar, Leiden Open Variation Database and Human Gene Mutation Database. Pathogenicity prediction scores were obtained for missense variants using SIFT, PolyPhen, MutationTaster and CADD. Polymerase chain reaction and Sanger sequencing for confirmation and familial segregation of the identified variants were performed on buccal swab‐extracted DNA.

The following variants were identified in the *MYH3* gene: c.2084G>T, p.(Arg695Leu) missense and 5′UTR, c.‐8‐2A>G, p.(?) (reference sequence NM_002470), without any other findings linked to DA or cleft palate. The panel used was the following from Panel App Australia: PanelApp_AUS/N47_Arthrogryposis_v0.414_GO, followed by an in‐house mendeliome for 4486 genes (see Supporting Information).

The missense variant is absent from the general population database (gnomAD v4.1) and from gene mutation databases (HGMD, LOVD, ClinVar). It is located within the *MYH3* motor domain, where pathogenic missense variants cluster. Prediction algorithms concur on the pathogenic nature of this variant.

The c.‐8‐2A>G 5′UTR variant is also absent from the general population database (gnomAD v4.1) and from gene mutation databases (HGMD, LOVD, ClinVar). Located in the intron 2 acceptor splice site, it is predicted to alter messenger RNA splicing (Alamut). The most likely consequence is a skipping of exon 3, which contains the start codon. There are no physiological transcripts with an alternative start codon, nor did we detect any other potential cryptic start codon for an optional 3′ translation.

Familial segregation study showed that both variants were present in the affected brother and absent in the unaffected brother. The 5′UTR splice variant was present in the mother and sister, neither of whom carried the missense variant. The latter was present in the father, who didn't carry the 5′UTR splice variant (Figure 1).

According to the American College of Medical Genetics and Genomics (ACMG) variant classification criteria [6], we classified both variants as likely pathogenic (class 4). The following criteria were respectively taken into account: PM1 (located in a mutational hotspot), PM2 (rare), PP1 (familial co‐segregation), PP3 (pathogenic bioinformatic predictions) and PP4 (specific phenotype) for the c.2084G>T variant; and PVS1_moderate (predicted null variant), PM2 (rare), PP1 (familial co‐segregation), PP4 (specific phenotype) for the c.‐8‐2A>G variant.

We returned the molecular result to the proband and her family, emphasizing the spectrum of clinical manifestations associated with known mutations in *MYH3*. In this case, the identified novel biallelic variants explain the arthrogryposis phenotype in the proband and her affected brother. The monoallelic 5′UTR splice variant may explain the mother and sister's phenotype, and the missense variant could also explain the father's subclinical manifestations appearing during physical training. The proband's pregnancy with a normal ultrasound monitoring was carried to term and her baby boy showed no signs of arthrogryposis. In this context and in agreement with the family, no prenatal diagnosis or presymptomatic screening for variants was performed. The study adhered to the Declaration of Helsinki and informed consents for genetic testing and publication were obtained.

## 3 Discussion

The first report of recessive *MYH3* variants in human disease was in 2018, describing four families with compound heterozygous variants and a spondylocarpotarsal synostosis phenotype, including cleft palate in one patient [4]. Biallelic *MYH3* variants were found in affected individuals in three of four families with unaffected parents. One of the alleles was a splice mutation (c.‐9+1G>A, reference sequence NM_002470) found in the Genome Aggregation Database (gnomAD v4.1.0) only in heterozygous individuals at an overall frequency of 0.001, but never in the homozygous state. It was functionally characterized as a hypomorphic allele, permissive of *MYH3* translational initiation but with reduced efficiency. The other *trans* alleles were different truncating variants. In the fourth family, where only one truncating allele, and no second allele, was found, the mother of two affected children interestingly showed bilateral camptodactyly of the fifth fingers. Hakonen and colleagues then reported in 2020 recessive *MYH3* variants in 4 patients from two unrelated families with an Escobar‐like phenotype and suggested the term “contractures, pterygia, and variable skeletal fusions syndrome 1B” [7]. Interestingly, the hypomorphic *MYH3* c.‐9+1G>A variant was reported as compound heterozygous with c.1053C>G; p.(Tyr351*) or c.3102+5G>C in the affected individuals. Novel truncating pathogenic variants were also reported in Caucasian patients all harboring the c.‐9+1G>A allele in *trans*. Clinical data showed significant overlap between patients with these recessively inherited variants and those with dominantly inherited *MYH3*‐associated disorders. The authors thus proposed the term “*MYH3*‐associated skeletal fusion” (MASF) syndrome as the same clinical entity with different inheritance patterns, possibly involving different pathophysiological pathways [3]. Finally, Morali et al. reported two pairs of *MYH3* rare homozygous missense variants in consanguineous siblings from a small family, presenting recessively inherited DA without any suggestive phenotype in heterozygotes [8].

Our report illustrates the complexity of *MYH3*‐related disorders and the need to expand the available classifications. Indeed, the proband's childhood clinical diagnosis of DA type 2B was refined by our molecular investigations to what can be considered a biallelic *MYH3*‐related disorder. To our knowledge, our observation is the second reported case of a biallelic *MYH3* transmission resulting in DA in two siblings, without any criteria for spondylocarpotarsal fusion or the proposed MASF nomenclature. In our case, careful examination leads to point out some minor abnormalities in heterozygotes, namely palatal cleft and/or congenital hip dislocation associated with the c.‐8‐2A>G variant in the mother and the sister, or muscle stiffness during exercise associated with the c.2084G>T variant in the father, which could fit with a slight effect of variants.

Further, this study underlines and confirms that variants affecting *MYH3* function can result in a very large spectrum of clinical manifestations with unclear genotype–phenotype correlations and various modes of inheritance. This finding has at least two important implications worth emphasizing. First, caution should be exercised when performing genetic testing in sporadic cases, as identifying a single variant—even one predicted to clearly affect *MYH3* function—may not be sufficient to fully explain the phenotype. Second, genetic counseling and recurrence risk assessment should be approached with particular care, as *MYH3* alleles implicated in *MYH3*‐associated disorders may exhibit dominant, recessive, or even co‐dominant inheritance patterns, especially in the presence of relatively frequent trans‐acting variants such as the hypomorphic c.‐9+1G>A allele.

Finally, our findings underscore that the nosology for clearly defining *MYH3*‐related conditions and their modes of inheritance in the clinical setting is still evolving, emphasizing the need to incorporate insights gained from careful analyses of both clinical and molecular data.

## Author Contributions

O.Z. and P.K.V.K. wrote the manuscript. O.Z., S.F., M.A., and P.K.V.K. followed the case clinically. O.Z. and P.K.V.K. contributed to the clinical and molecular diagnosis, as well as familial segregation studies. T.R.‐F., M.G., and T.N. performed and interpreted molecular analysis. All authors contributed to the manuscript elaboration and reviewed it critically. P.K.V.K. supervised the manuscript.

## Funding

The authors have nothing to report.

## Conflicts of Interest

The authors declare no conflicts of interest.

## Supporting information


**Data S1:** cge70142‐sup‐0001‐Supinfo.xlsx.

## Data Availability

The data that supports the findings of this study are available in the Supporting Information of this article.
